# Pituitary Destruction by Injection of Radioactive Substance and Section of the Pituitary Stalk for Advanced Cancer

**DOI:** 10.1038/bjc.1957.2

**Published:** 1957-03

**Authors:** F. L. Davies, P. H. Buxton

## Abstract

**Images:**


					
8

PITUITARY DESTRUCTION BY INJECTION OF RADIOACTIVE

SUBSTANCE AND SECTION OF THE PITUITARY STALK FOR
ADVANCED CANCER

F. L. DAVIES AND P. H. BUXTON

From the Neurosurgical Unit and the Bland-Sutton Institute of Pathology,

Middlesex Hospital, London, W.1.

Received for publication January 30, 1957

TRANSFRONTAL hypophysectomy is an established surgical procedure in the
treatment of advanced cancer. Results of this operation have been reported
by Perrault, Le Beau, Klotz, Sicard and Clavel (1952), Shimkin, Boldrey, Kelly,
Beirman, Ortega and Naffziger (1952), Luft and Olivecrona (1953) and by Pearson
Ray, Harrold, West, Li, Maclean and Lipsett (1956).

Luft and Olivecrona (1953) performed hypophysectomy in 15 cases of malig-
nant disease. Twelve cases suffered from carcinoma of the breast, one from chorion
carcinoma, one from carcinoma of the kidney, and one from carcinoma of the
prostate. They showed that when hypophysectomy is complete a remarkable
regression of the carcinoma may occur in cases of metastatic breast cancer. There
were no operative deaths in this series.

Pearson et al. (1956) have reported a series of 79 patients with advanced malig-
nant disease on whom hypophysectomy had been performed from 1953-55. There
were 11 operative deaths in the whole series. Forty-one patients with metastatic
breast cancer could be evaluated and 21 of these obtained objective remission.

All the available evidence indicates that only those cases of breast cancer
which are hormone dependent benefit from removal of the pituitary. The present
difficulty in selecting patients with breast cancer for operation is to determine
those whose growths are in fact hormone dependent.

Two possible tests are available. In one (Pearson et al., 1956) oestrogens
are given and the effect on the mammary cancer noted, an increase in activity
indicates that the growth is influenced by oestrogens and that hypophysectomy
might be of value. The second test is the estimation of the mammatrophins in
the urine (Hadfield, 1956). Further work on these methods is awaited with interest.
It should be noted however that while patients who have had a favourable
response to oophorectomy or to androgen therapy are likely to have a favourable
response to hypophysectomy, failure to respond to androgen or oestrogen therapy
does not necessarily mean that the patient will fail to respond to hypophysectomy.

In view of the reported results of hypophysectomy in advanced cancer, it
was decided to attempt to destroy the pituitary gland by local irradiation in
cases of malignant disease where the tumour was likely to be hormone dependent.
Chromic phosphate was injected into the gland, the phosphorus being in the form
of the radioactive isotope p32. This method was described by Rothenberg, Jaffe,
Putnam and Simkin (1955). It appeared a less severe procedure than hypophy-
sectomy in a seriously ill patient.

Radioactive colloidal chromic phosphate seemed theoretically an ideal sub-
stance for pituitary ablation by radiation. It is precipitated upon injection and

PITUITARY DESTRUCTION FOR ADVANCED CANCER                9

remains at the injection site; p32 emits beta radiation only with a maximum
effective penetration of 0.8 mm. in body tissue; . it has a half-life of 14 days which
is very convenient when planning the operation. Radioactive gold does not
remain at the injection site and it has a half-life of 31 days, thus like radioactive
yttrium (half-life 61 hours), it has to be obtained immediately before operation
to get a sufficiently high dose of radioactive substance in a small volume of the
injection material.

Pituitary Destruction by Injection of Radioactive Chromic Phosphate

Between October 1955 and June 1956, 5 cases of advanced carcinoma of the
breast with widespread metastases and 1 case of malignant melanoma with meta-
stases were treated by injection of radioactive chromic phosphate into the pituitary
gland. These 6 cases form Group I (Table I). The effectiveness of the procedure
was assessed by eridocrine and electrolyte changes.

TABLE I.-Summary of Cases Treated for Advanced Carcinoma

Group I

Cases treated by injection of radioactive chromic phosphate

Site of

Sex          primary tumour
F.      .        Breast
,,      .        Skin

(malignant melanoma)
.. .             Breast

.. *     *            .    .    9
..9      *            * .  .

.. *1    *            .    .    9

* Stalk section carried out 23 weeks after injection.

Post-operative

survival

Died at 7 weeks

.   ,,  26  ,,

10
30
29
22

Group II

Cases treated by injection of radioactive material, and section of pituitary stalk

Sex
F.

Site of

primary tumour

Breast

Post-operative

survival

Died at 30 weeks

50       .        .         , .                           ,,  ,,  4    ,,

52       .                .                         .      ,,  ,,  9 days.
In Groups I and II operation produced no appreciable effect on the tumour.

Group III

Cases treated by section of the pituitary stalk

Age
57

oQ

Sex
F.

Site of

primary tumour

Breast

Effect on tumour

Relief of bone pain

LU    .     on    .    ss    .      ,,     *      2X      ,

11    .    42     .          ,,  .  ,,     . Regression of cuta-

neous and pleural
deposits

12    .    63     .    M.    .  Prostate   . Marked fall in acid

phosphatase
13    .    46     .    F.    .   Breast    . No change
14    .    42     .    ..    .      ...       ..    ..

Post-operative

survival

Alive at 21 weeks.
Died at 14    ,,
Alive at 13   ,,

Alive at 12   ,,

Died at 10    ,,
Alive at 5     ,,

Age
42
28

Case

1
2
3
*4

5
6

36
47
54
40

Case

4

8
8

Age
47

Case

9

,,9

,,~
,,9
,,9
,,9

F. L. DAVIES AND P. H. BUXTON

Investigations

The following tests of pituitary function were carried out before and after
operation: estimation of the basal metabolic rate, the radioactive iodine uptake
and excretion, the urinary 17 ketosteroids (17 KS) and glucocorticoids (17 OH).
The change in excretion of the glucocorticoids was found to be of more significance
than the change in the ketosteroids in assessment of adrenocortical function,
as ketosteroid output is always low in seriously ill patients. A complete radiological
survey was undertaken and estimations of the 24-hourly urinary calcium excretion
were made. A full blood count was done and the blood urea, serum cholesterol,
blood sugar, plasma proteins and electrophoretic pattern and serum electrolytes
were estimated. The most helpful of all these tests were found to be the radio-
active iodine uptake and excretion and the glucocorticoid estimation.

Operative procedure

This has been previously described (Davies, 1956). In summary, a coronal
scalp incision is made, the flap being turned down over the forehead and eyes.
A trephine opening 1 -in. diameter is made above the superior orbital ridge, the
frontal lobe is elevated, the olfactory tract is cut and the pituitary stalk is seen
below the optic chiasm. After injection of the radioactive material the bone disc
is replaced and the scalp sutured in the usual way. The amount of radioactive
material injected varied from 3-8 millicuries in 0.2-0-4 ml. of solution.

Post-operative complications

All patients complained of headache and a feeling of fullness behind the eyes
for a few days, aspirin or codeine tablets were sufficient for the relief of these symp-
toms. Somnolence was noted in all patients but in only 1, Case 6, was cortisone
necessary for relief. One patient developed a temporary right temporal field
defect which cleared up in 10 days. Another patient complained of mistiness of
vision which disappeared in 14 days.

Results

In the first 5 cases there was no clinical nor endocrine evidence of effective
pituitary destruction. In Case 6 however there was profound disturbance soon
after operation (Fig. 1). In this case there had been some haemorrhage in the
pituitary region at operation and it was thought that the pituitary stalk might
have been damaged whilst controlling this bleeding.

Autopsy was possible only in Case 2 of this group. Widespread tumour
deposits were found in this case. The pituitary gland showed partial destruction
of the epithelial elements (Fig. 2) but viable glandular tissue remained. The
hypothalamus showed nodules of tumour cells but no gross parenchymal damage
(Fig. 3, 4). The thyroid and suprarenals though extensively infiltrated by carci-
noma showed no gross damage to secretory tissue.

In view of these findings and the absence of endocrine changes in life in the
cases other than Case 6, it was decided to combine injection of radioactive chromic
phosphate with deliberate destruction of the pituitary stalk. Three cases were
treated in this manner and form Group II (Table I). Pre- and post-operative

10

PITUITARY DESTRUCTION FOR ADVANCED CANCER

investigations were done as before. The technique of the combined operation
was as described above but after the gland had been injected the pituitary stalk
was divided close to the gland and the ends cauterised. (Fig 5).

CASE6

I0
/~o

Active 5
/odge

44

.3

Adrenal
Shtod

(xcretion  2
()I/0        ?

74 1          M9X  /X   ISIS  /  /5  2 %   45

DA FE

FIG. 1.-Case 6. Post-operative fall in radioactive iodine uptake and fall in "17 OH"

excretion. Reversal of these changes after thyrotrophic and adrenotrophic hormone is
also shown.

Pituitary Destruction by Injection Combined with Stalk Section

The first two cases treated by this combined method showed marked endocrine
changes (Fig. 6). As Case 4, treated earlier by injection only, still showed no
endocrine disturbance, nor was there any alleviation of symptoms from malignant
disease, she was operated upon again and stalk section was performed. Immediately
there were profound endocrine changes (Fig. 7). There was however no appreciable
change in the patient's extensive malignant disease, the local skin deposits practic-
ally surrounded the chest.

Autopsy was possible in Cases 7 and 8 treated by the combined method.
In Case 7 gross pituitary destruction was found though a small island of viable
epithelial cells remained (Fig. 8). The hypothalamus in this case showed very

%  Abw uptoke

% ,, How,pro,o,

%\\\i Nhb e,reion

10

C0m

0o                        L17OH'
ro

o0                                            i

11

I

F. L. DAVIES AND P. H. BUXTON

marked parenchymal damage which was thought to be due to ascending throm-
bosis from the cauterised, divided pituitary stalk (Fig. 9). Small groups of tumour
cells were also seen in the hypothalamus (Fig. 10). The thyroid showed no gross
histological abnormality; the suprarenal glands had been removed previously.
In Case 8, despite the marked endocrine changes found in life, autopsy showed
only partial destruction of the pituitary epithelial tissue, no gross hypothalamic
damage, but complete necrosis of the distal part of the pituitary stalk (Fig. 11).
The thyroid and suprarenal cortex showed degenerative changes and extensive
infiltration by carcinoma cells.

CA4SE7

/00-

,q'aUooctAv

bdhe50-

0

June           30        /0       202
June       9    ~~~~~Ju~y K)

FIG. 6.-Case 7. To show post-operative fall in radioactive iodine uptake.

These results suggested that destruction of the pituitary stalk produced
profound endocrine disturbance despite incomplete destruction of the pituitary
glandular tissue, whereas incomplete destruction of the glandular tissue alone
produced no such effect. It was therefore decided to treat the next group of
cases by destruction of the pituitary stalk alone without injection of radioactive
material. Support for this method of treatment was obtained by reference to
the publication of Professor D. S. Russell in which the effects of dividing the
pituitary stalk in man were discussed (Russell, 1956).

Pituitary destruction by stalk section alone

Six patients have been treated by section of the pituitary stalk alone, and
form Group III (Table I). Five of these were cases of carcinoma of the breast,
the other was a case of carcinoma of the prostate.

The pre- and post-operative investigations were similar to those employed
in Groups I and II and the same operative technique was used.

~%NM h*xr 4roke
ESE0%,hburom0ik,

I

i

ct

}X            ~~~~~~~~~~~~~~~~~~to

ii            I

12

I

PITUITARY DESTRUCTION FOR ADVANCED CANCER

Post-operative progress

In all cases of Group III there was immediate hypothermia which was followed
in 12 hours by pyrexia of 100? to 102? F. which persisted for 48 hours. Somnolence,
tachycardia, and hypotension developed within 12 hours, but were abolished
by intravenous hydrocortisone. A maintenance oral dose of hydrocortisone was
attained in 2 weeks. Case 14 was given pre-operative intramuscular cortisone
and did not develop these symptoms.

CASE 4

%04 Hour ptoke

~~~~.g                      ~ !~ ~ ~~~~~1%z4 Htou excretilo

A' ptake suAeresued
/00                                            by thyroid thero,

Radio

Active 50 *O
Iodine

0~~~~~~~

I

0 41/                               1OH'                I

6           71 ';        '7K-S|

F2- -~

A ,e ,

steroid/h                      hah     ^  Dh5      3

FIG. 7.-Case 4. To show post-operative fall in" 17 OH "excretion and complete suppression

of radioactive iodine uptake and excretion till start of steroid therapy.

Two patients had a generalised convulsion 7 days post-operatively, the fits
were controlled by intramuscular phenobarbitone.

In 4 patients there was an immediate polyuria after operation which persisted
until injections of pitressin tannate were given at weekly intervals.

Headache was present in all cases but usually disappeared completely in
4-5 days.

The radioactive iodine uptake was considerably reduced in all cases post-
operatively. In 1 case this occurred as early as the third post-operative day.
In Case 11 the 1131 uptake fell from a pre-operative value of 43 per cent to 7
per cent while in Case 12 the values were 31 and 3 per cent respectively. The
urinary 17 OH and 17 KS rapidly decreased in all cases post-operatively. Repre-

13

F. L. DAVIES AND P. H. BUXTON

CASE 9

/00-
Z/z

%N hbw

5O.

Excre6bn

20

mg/day

10-

0

DArF

FIG. 12 (Case 9), FIG. 13 (Case 10).-To show post-operative fall in radioactive iodine uptake

and steroid excretion.

sentative results of radioactive iodine and steroid excretion are shown in Fig. 12
and 13.

In Case 12, a man with skeletal metastases from prostatic carcinoma, the 24-
hour urinary calcium excretions pre-operative values were 42, 52, 36, 42 mg.
per 100 ml. The post-operative values were 14, 19 and 24 mg. per 100 ml. These
figures suggested marked reduction in activity of the bony metastases. In this
patient also there was a dramatic fall in the acid phosphatase after operation
(Fig. 14).

Cases 9 and 10 had been bedridden with pathological fractures of the femora
before operation. They were improved to the extent of being able to walk out of
hospital after operation. In both these cases and in Case 12 there was marked
relief of bone pain and subjective improvement, but despite this there were no
changes in the radiological appearances of the skeleton.

Case 11 had multiple secondary deposits in the lung with a pleural effusion.
She showed remarkable improvement after operation. Her pleural effusion which
had required aspiration before operation did not recur; the lung deposits and
the cutaneous metastases (Fig. 15) showed regression. There was no improvement
in the cutaneous deposits in Case 14, 5 weeks after operation; Case 13 died 10
weeks after operation without change in her malignant disease.

All the cases in this group claimed marked improvement subjectively.

I              _sz

al-           % ~~~? 24 hh urexcretion

4~~~~

ts

1/// 7 K-S'

1S~~~ hLr> I      l D - "

14

15

PITUITARY DESTRUCTION FOR ADVANCED CANCER

CASE /0

-        Uptoke

I>1S\\\   Excretion
a

4  /I       /3   18     4    26    12    19    2d         ,,

Sep                       Oct

7

Operotion

~~~~~~~~~~~~~~~~~~']''/-/-'   i I-  i ,
stE/kn    +v               I ,       [I       1

77,     /7 7 c

L MJi5

I1

l ELmi.A

.11  f   It-  . 0 ,4  ).  to* Alk  Iafne  IVJdu

Sp. ,A56
FIG. 13.

_ |  X----X AlkoinePhosph

/,x

/ x

I   I   I( H/

I   I   I            _J I   I

0

CASE 12

*     * Acid Phosphotase

atase

-/0  -        5   O    /,S  20  25

IDoy~
Operotion

FIG. 14.-Case 12. To show changes in acid and alkaline phosphatese after stalk section in a

case of carcinoma of prostate.

AO

Jl

0
~0
0.

ro
'0
0

17 QH
7 S.Sa
MAy

A

60
70

60

~so

. 40
sK

20
I0

-                   - ?                                    I   1

9    /n   /i

1             2      Ly     Ad     -V     /J      /7   /o        in     A

_-W

I/ K. lb.

h        ,^      I

p  w #& '.. .

F. L. DAVIES AND P. H. BUXTON

CONCLUSIONS AND SUMMARY

Fourteen cases of advanced malignant disease were treated by methods
designed to destroy pituitary glandular tissue. In Group I, 6 cases, the pituitary
gland was injected with radioactive chromic phosphate. Complete destruction
of the gland was not attained in any case, nor was any improvement seen in the
malignant disease for which the patients had been treated. The only case which
showed any endocrine disturbance was Case 6 and at operation the pituitary
stalk of this patient had been damaged.

In Group II (3 cases, 1 of which had already been treated unsuccessfully in
Group I) and Group III (6 cases), the pituitary stalk was divided. Immediate
and profound endocrine disturbance was evident, though the two autopsies
performed on cases inl Group II showed viable pituitary epithelial tissue still
present. There was no apparent difference between cases treated by stalk section
alone and those treated by stalk section and injection of radioactive material.
Suibjective improvement and relief from pain was claimed by all patients treated
by stalk section and in 4 cases there was objective evidence of improvement.

It is felt that the technique of operation described for section of the pituitary
stalk through a trephine hole without a major craniotomy is a relatively simple
procedure; it has proved safe in our hands even when used for seriously ill patients.
It is for this reason that we decided to publish this small series at this time. It is
appreciated that, though this procedure has been shown to produce severe endo-
crine changes suggesting suppression of pituitary glandular function, our longest
follow-up is only 21 weeks after stalk section, so we do not yet know how permanent
the endocrine changes may be. If pituitary function should recover at a later
date due to re-growth of pituitary glandular tissue, this might be prevented by
injection of radioactive material into the gland at the time of stalk section using
the same operative approach.

The outstanding problem is the selection of cases of malignant disease in which
regression of the tumour may be expected as a result of the severe endocrine
changes produced by suppression of pituitary function.

EXPLANATION OF PLATES

FIG. 2.-Pituitary gland-Case 2. Showing partial destruction surviving tissue in left upper

quadrant. H. & E. x 2-5.

FIG. 3.-Hypothalamus Case 2. Showing nodule of tumour cells. H. & E. X 10.
FIG. 4.-As Fig. 3. H. & E. x 50.

FIG. 5.-To show operative approach.

FIG. 8. Pituitary gland-Case 7. Showing almost complete destruction. There is a small

island of viable cells at the left lower border. H. &. E. x 3.

FIG. 9. Hypothalamus-Case 7. Showing marked destruction. H. & E. x 10.

FIG. 10.-High power view of group of tumour cells just above centre of Fig. 9. H. & E.

FIG. I 1.-Pituitary gland and hypothalamus Case 8. Showing (a) destruction of distal

part of stalk, with thrombosis of portal vessels; partial destruction of gland-surviving
tissue left lower quadrant. H. & E. x 5. (b) Higher power view of surviving glandular
tissue. (c) Higher power view of pituitary stalk.

FiG. 15.-Case 11. To show regression of cutaneous metastases 2 months after stalk section.

16

BRITISH JOURNAL OF CANCER.

2                         8

3

9

4                                    10

Davies and Buxton.

Vol. XI, No. 1.

A

CANCER OF THE LUNG IN RELATION TO TOBACCO

of this factor, but in this country (Stocks, 1936; Kennaway and Kennaway,
1947) and in Norway (see above) the mortality is higher in towns than in the
country. In all such comparisons one must consider at least three possible
factors, namely, (a) smoking habits, (b) facilities for diagnosis and treatment, and
(c) atmospheric pollution with products of the combustion of coal tar, or of the
internal combustion engine.

(5) The indication that cigarettes are more active than cigars and pipe tobacco
in relation to cancer of the lung raises the question, whether this difference is due
(a) to the method of combustion, or (b) to some property of cigarette tobacco. One
cannot answer this question at present, but it is especially important in countries
where smokers buy the cheaper pipe tobacco to make their own cigarettes. In
Norway one obtains in this way nearly twice as many cigarettes for a given sum
(Jakobsen, personal communication). One must consider three possibilities: (1)
Pipe tobacco smoked in pipes; (2) pipe tobacco smoked in cigarettes; (3) cigar-
ette tobacco smoked in cigarettes.

(6) The data given and discussed above from this country, Norway and
Switzerland show that the study of the relations of national consumption of
tobacco, and national incidence of cancer of the lung, has scarcely begun.

SUMMARY.

(1) Estimations of arsenic in cigarettes from the United States, Canada,
England, Norway, France, Switzerland, Italy, Austria, Poland and Bulgaria show
on the whole a transition from the arsenic-rich American type in the West, to the
arsenic-poor Turkish type in the East; the latter is, of course, smoked in Western
countries also.

(2) In Yugoslavia, Turkey, Greece and Bulgaria, the tobacco consumed is
almost wholly home-grown, of Turkish type, and in the form of cigarettes.

(3) The high incidence of cancer of the lung at autopsy in one centre (Istanbul),
in a country where Turkish tobacco is smoked almost exclusively, shows that the
arsenic content of tobacco has not provided any simple and exclusive explanation
of the association between cigarette smoking and this form of cancer. Cancer
of the lung appears to be much less frequent at a centre in Yugoslavia (Ljubljana)
than at Istanbul. More information from these and other Balkan countries is
very desirable.

(4) Norway imports about 85 per cent of the tobacco consumed from the
U.S.A., and 9 per cent from the Balkan countries. The incidence of cancer of
the lung upon the two sexes is not very different (death rate, male to female,
1: 0 7); it is greater in urban than in rural districts, and this difference is greater
in men than in women. The increase in mortality in the last 20 years has been
greater in men than in women, and greater in the towns than in the country.

(5) In Sweden, snuff makes up a much larger fraction (one-third in 1949) of
the total tobacco products consumed than is recorded in other countries.

(6) A comparison is made of the data available for the increases since 1931 in
(a) deaths attributed to cancer of the lung, and (b) in the consumption of tobacco,
in England and Wales, Norway and Switzerland. The consumption of tobacco
per head has been for the last 10 years rather higher in Switzerland than in the
United Kingdom, and in Norway has been about one-half that in the other two
countries, while the crude death rates at the beginning and end of the period were

19

BRITISH JOURNAL OF CANCER.

lib

Ila

lic

Davies and Buxton,

Vol. XI, No. 1.

I

%i-.                             I     I   ..

6- 'O.

0

*- e~

6
z

-
0

0
z

0
z

9
oD

q

PITUITARY DESTRUCTION FOR ADVANCIED CANCER    17

We wish to thank Dr. J. D. N. Nabarro and Dr. G. Walker who have collabor-
ated in this work and undertaken the metabolic and endocrinological investigations
which will be the subject of a later report.

Dr. A. M. Jelliffe and Mr. R. E. Ellis collaborated in the work in which radio-
active chromic phosphate was used.

Our thanks are also due to Professor B. W. Windeyer, Miss M. D. Snelling,
Mr. D. H. Patey and Mr. R. S. Handley for their co-operation and permission
to operate on their cases.

We are grateful to Mr. T. E. Cowan for drawing the charts and to Miss E.
Hewland and Mr. M. Turney for the drawings and photographs.

Part of the expenses of this investigation were met by a grant from the British
Empire Cancer Campaign.

A paper based on the earlier cases in this report was read before the Society of
British Neurological Surgeons in November 1956.

REFERENCES
DAVIES, F. L.-(1956) Lancet, i, 466.

HADFIELD, G.-(1956) Brit. med. J., i, 94.

LUFT, R. AND OLIVECRONA, H.-(1953) J. Neurosurg., 10, 301.

PEARSON, O. H., RAY, B. S., HARROLD, C. C., WEST, C. D., Li, M. C., MACLEAN, J. P.

AND LIPSETT, M. P.-(1956) J. Amer. med. Ass., 161, 17.

PERRAULT, M., LE BEAU, J., KLOTZ, B., SICARD, J. AND CLAVEL, B.-(1952) Therapie,

7, 290.

ROTHENBERG, S. F., JAFFE, H. L., PUTNAM, T. T. AND SIMKIN, B.-(1955) Arch. NTeurol.

Psychiat., Chicago, 73, 193.

RUSSELL, D. S.-(1956) Lancet, i, 466.

SHIMKIN, M. B., BOLDREY, E. B., KELLY, K. H., BIERMAN, H. R., ORTEGA, P. AND

NAFFZIGER, H. C.-(1952) J. cdin. Endocrin., 12, 439.

ADDENDUM

Since this paper was submitted for publication Cases 9 and 12 in Group III have
died, 6 and 5 months, respectively, after operation. Section of the pituitary glands
in both these cases at autopsy has shown surviving pituitary glandular tissue
amounting to approximately 20 per cent of normal, mainly around the periphery
of the gland. There is no evidence in these cases of functional reconnection of the
pituitary gland to the hypothalamus nor of re-growth of glandular tissue around
the divided stalk.

2

				


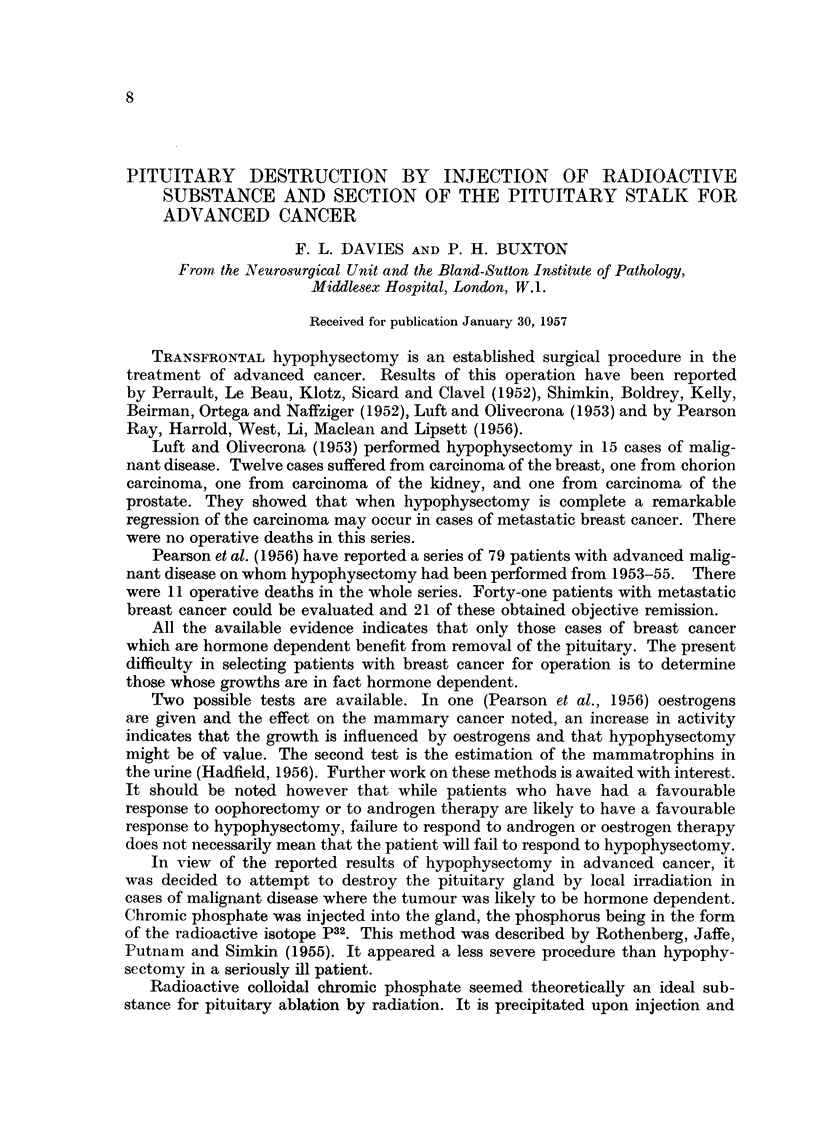

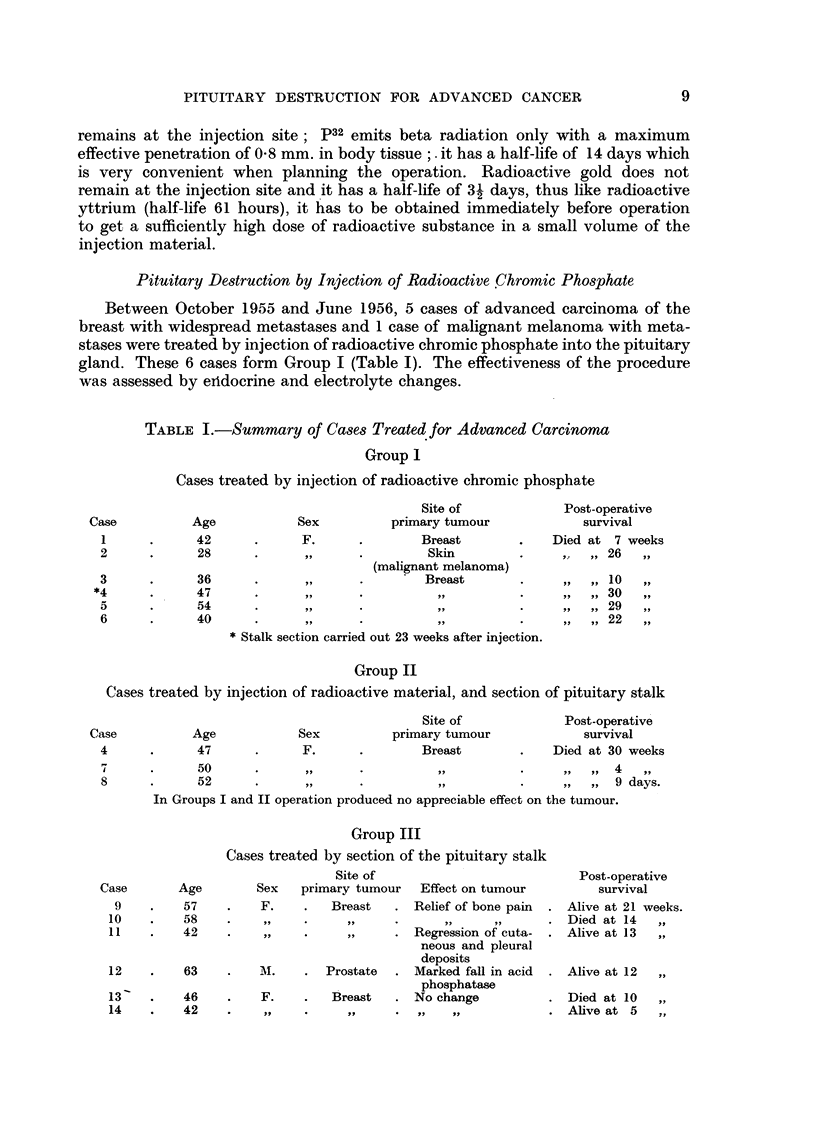

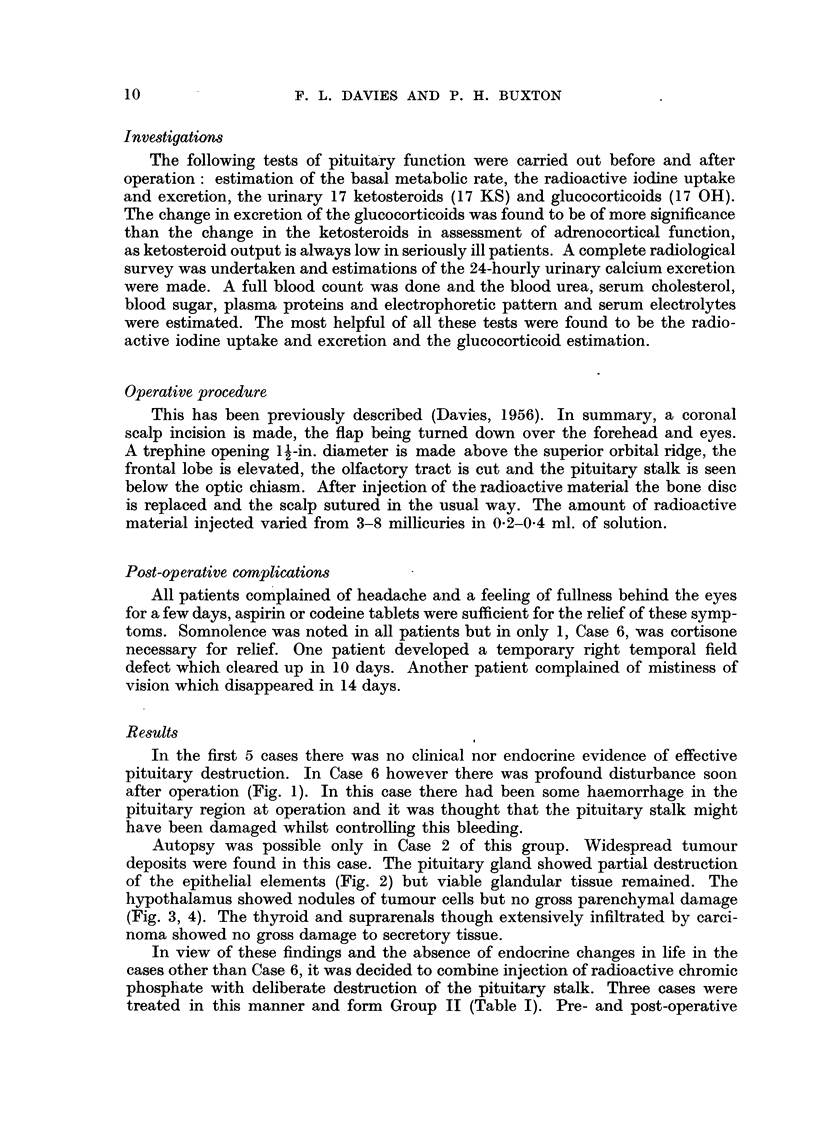

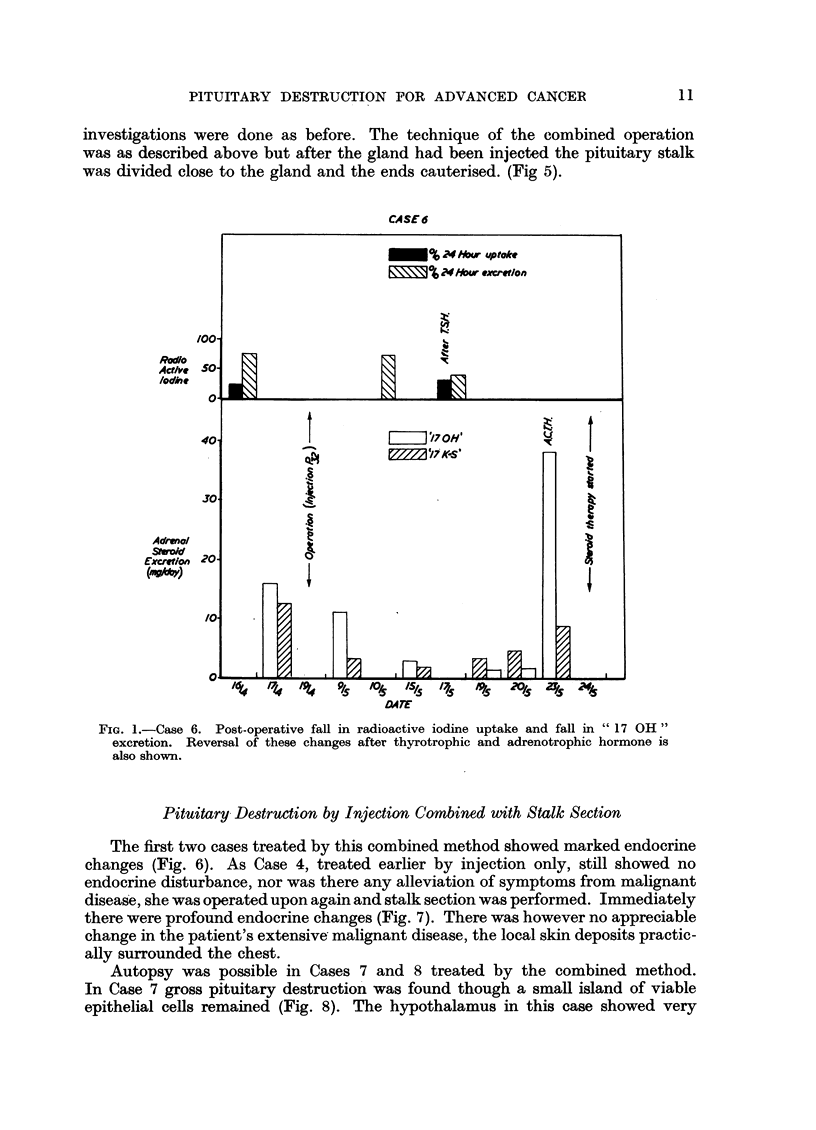

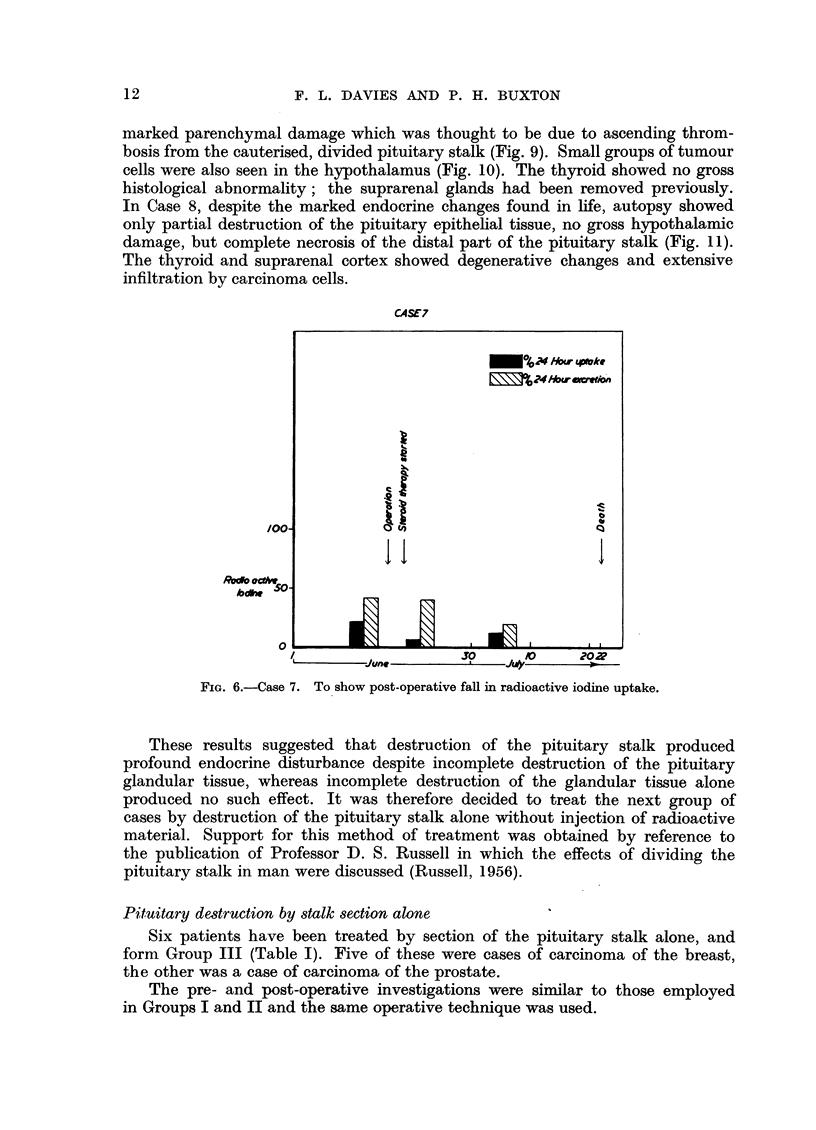

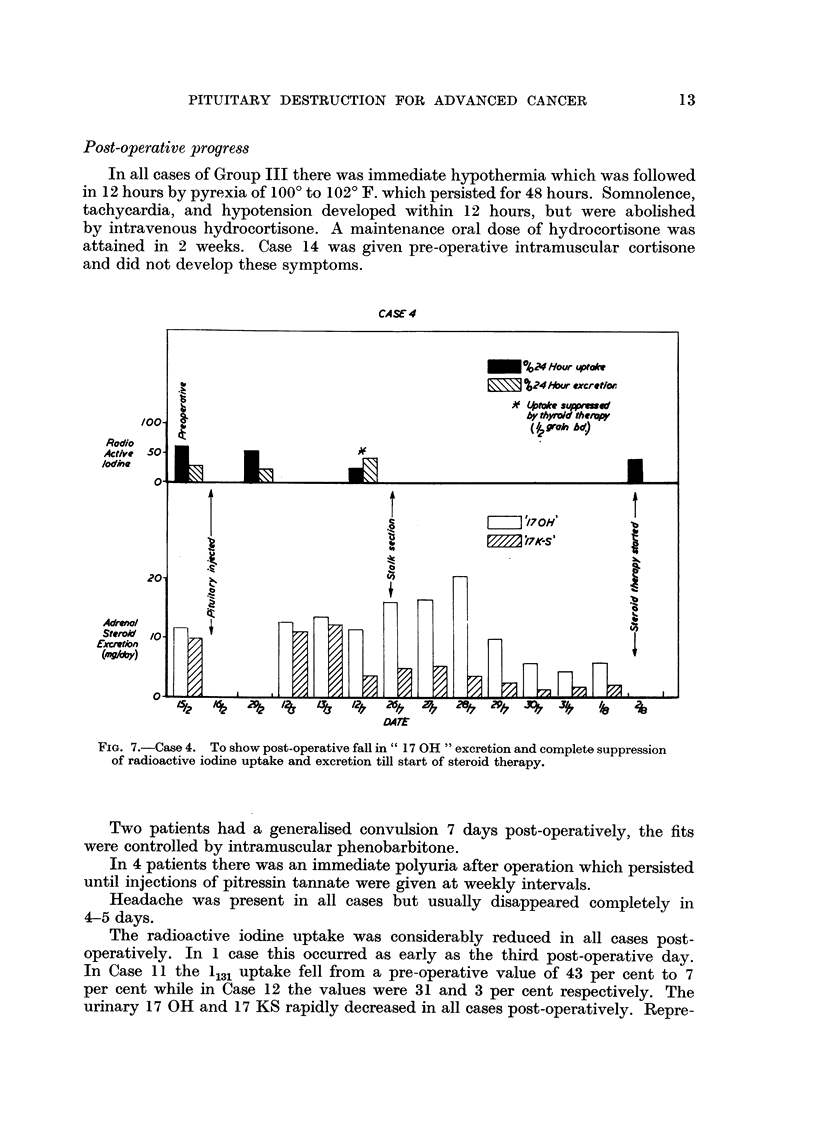

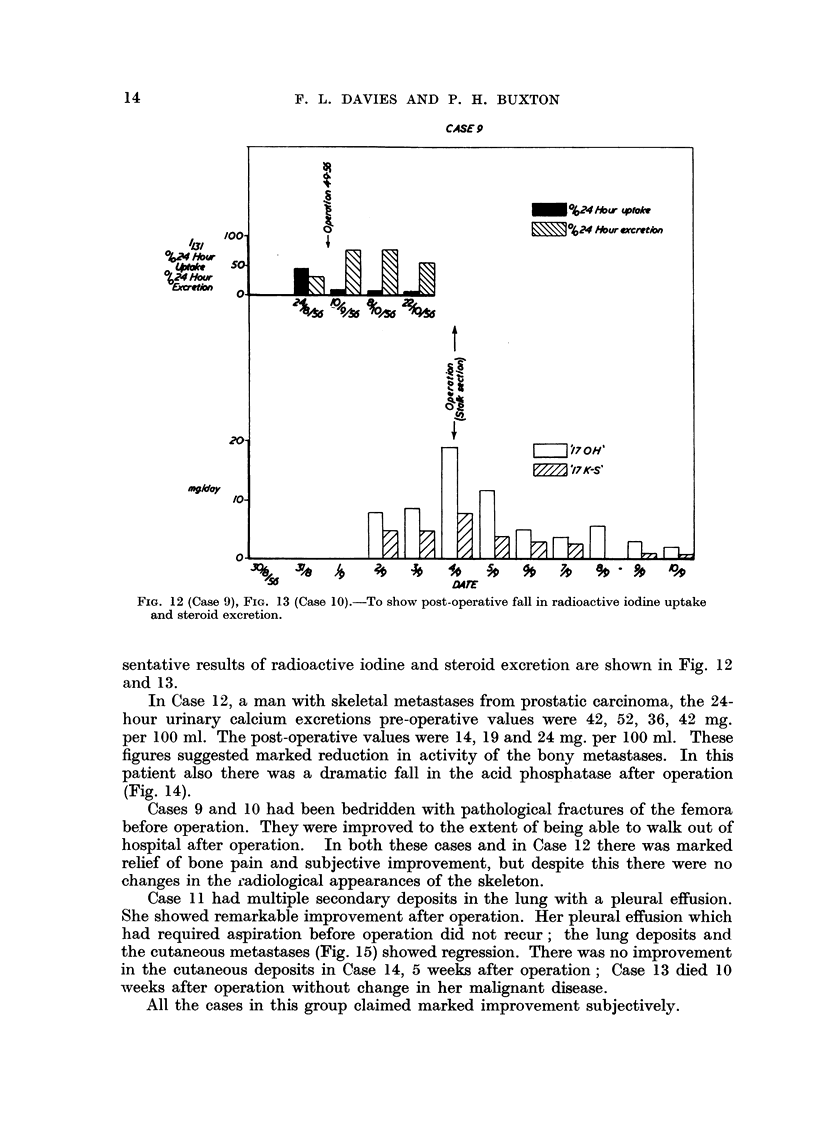

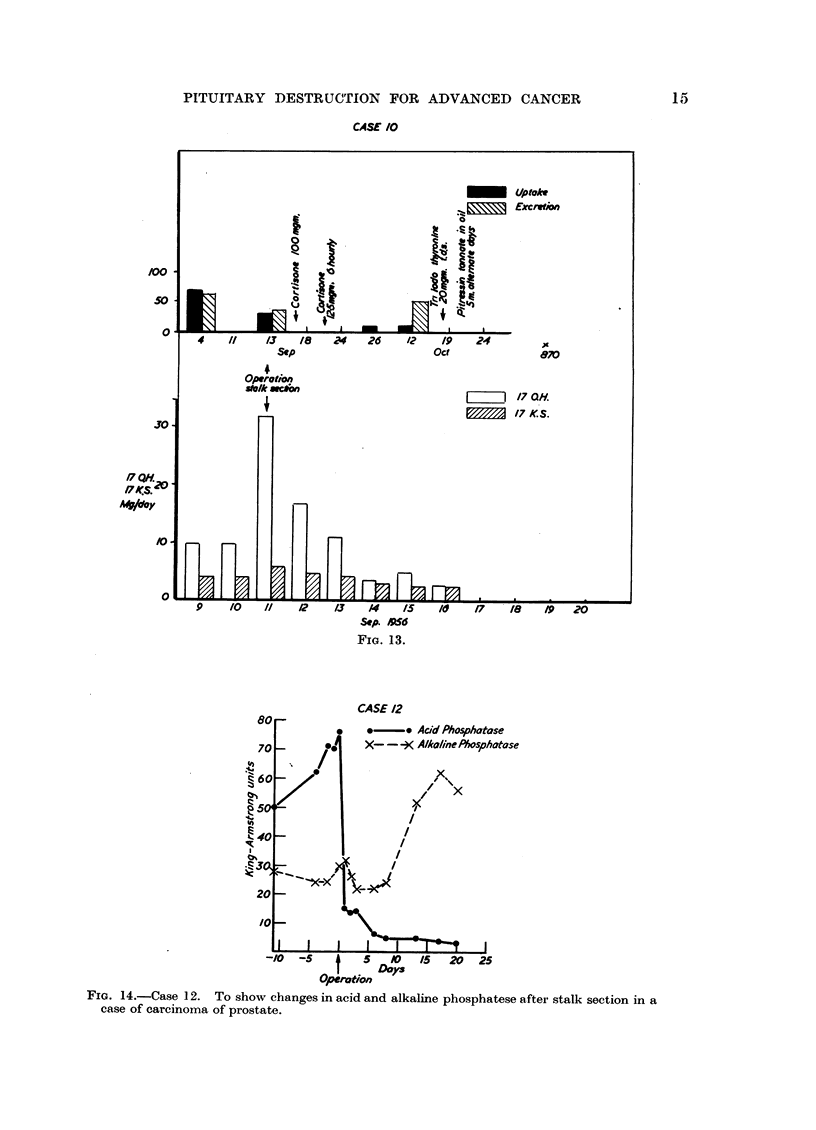

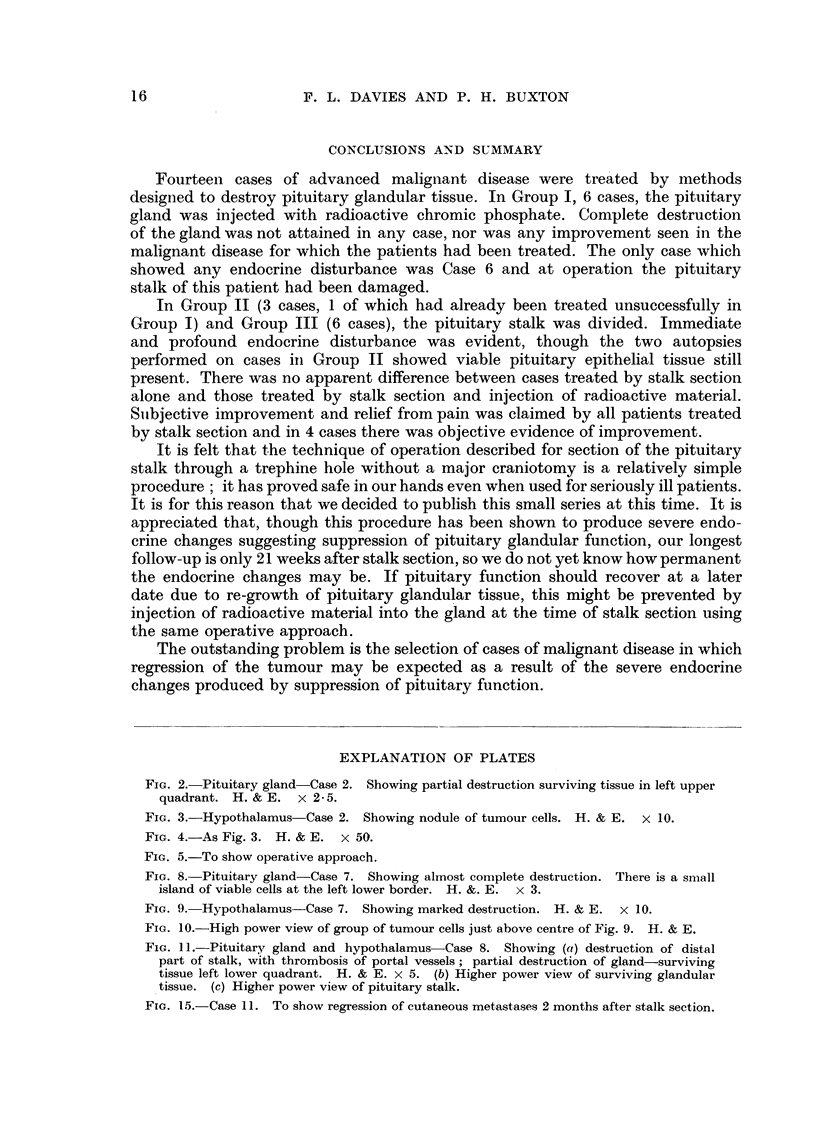

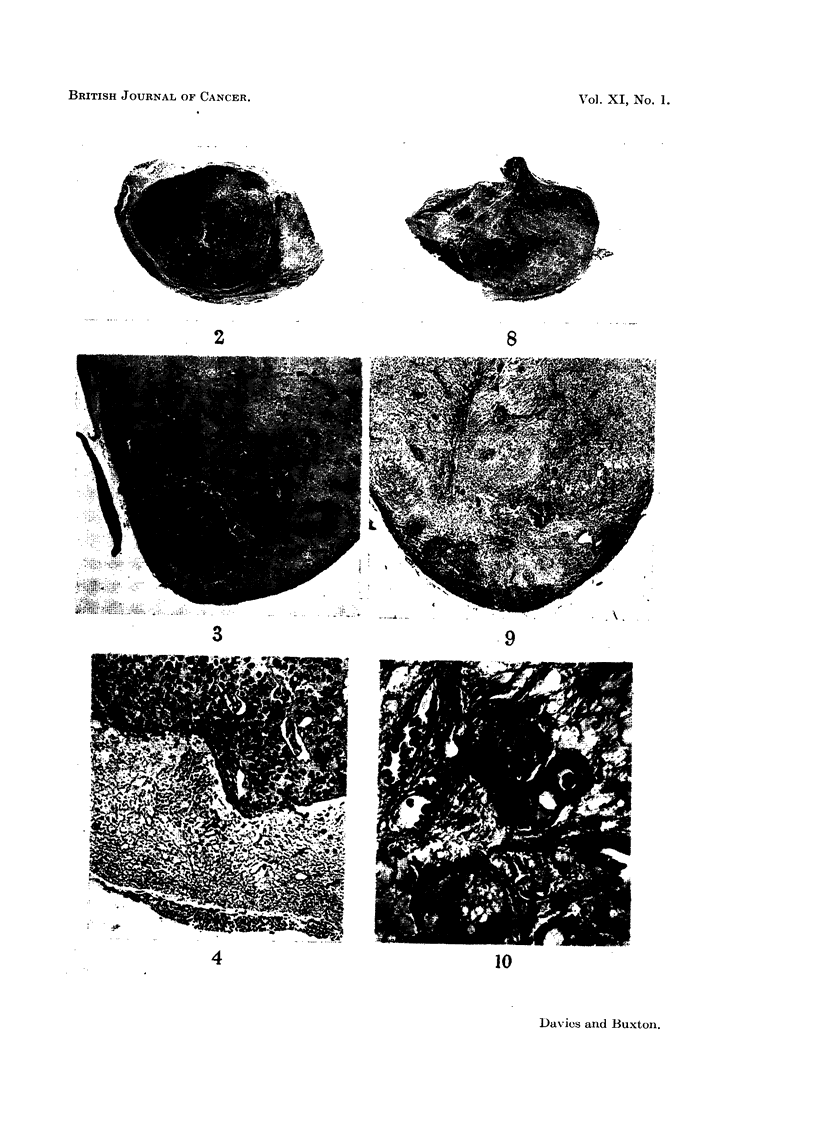

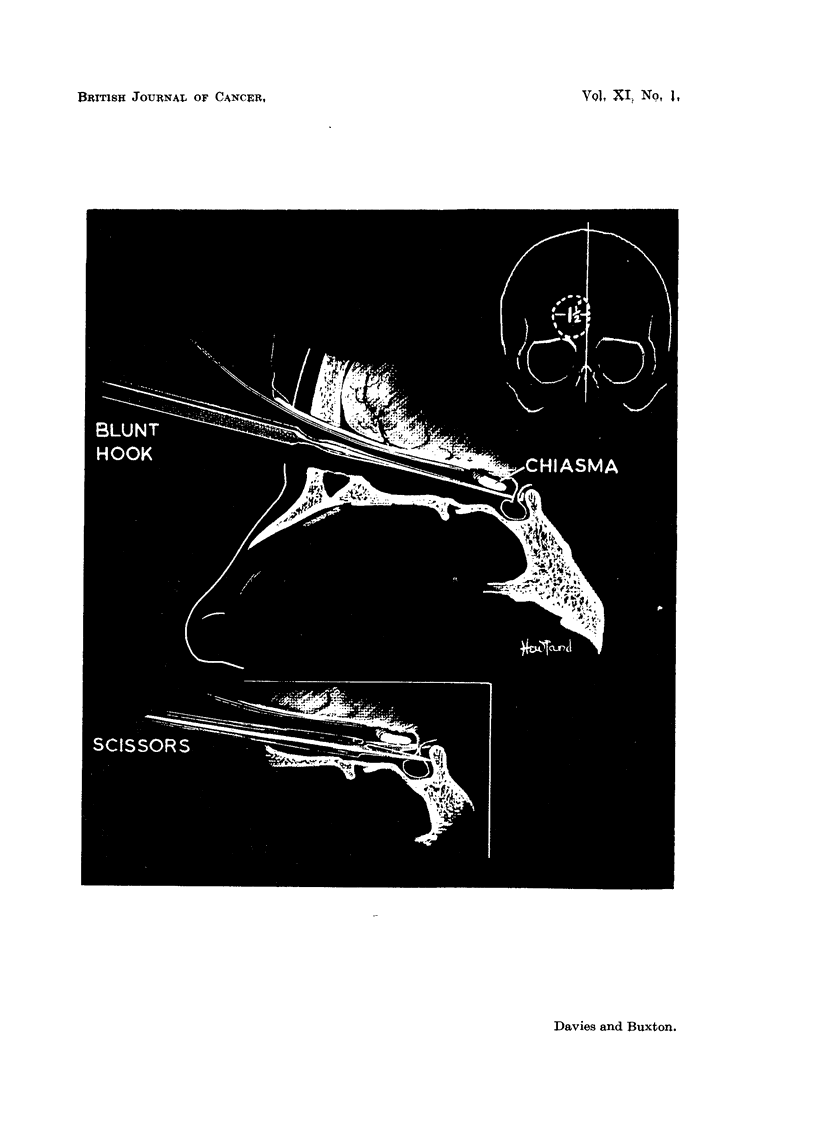

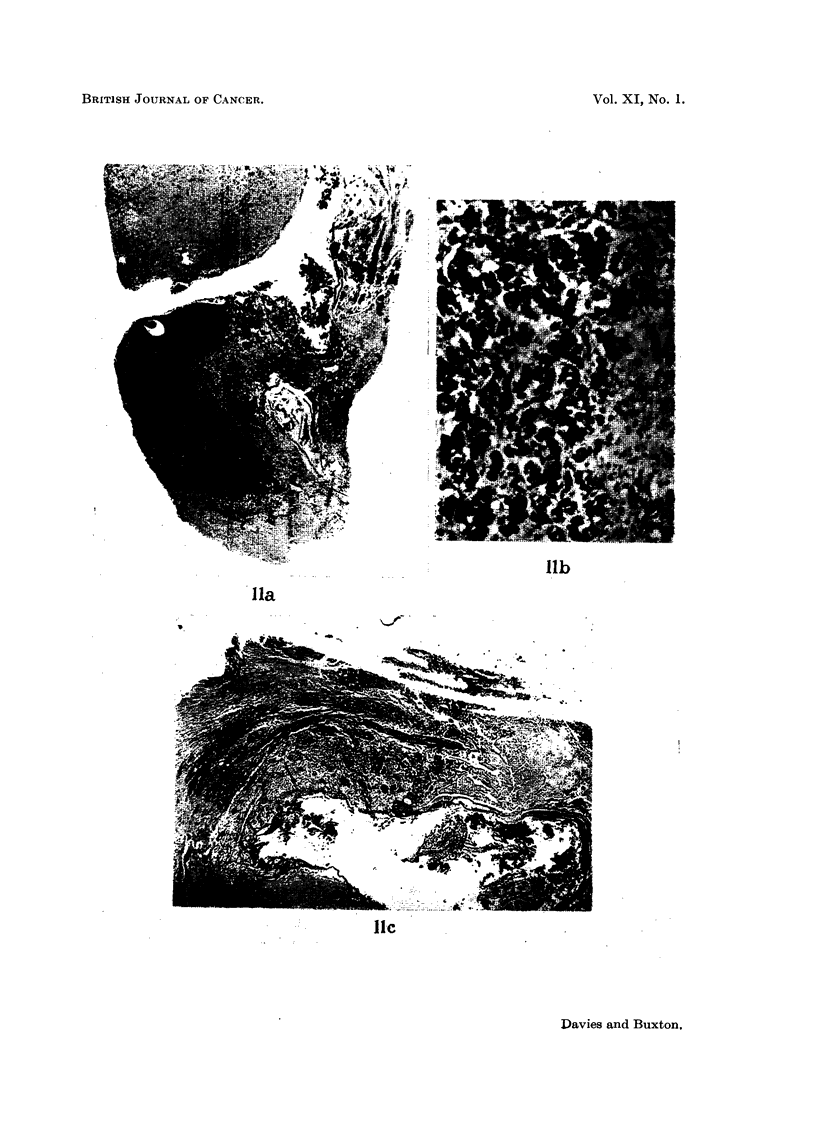

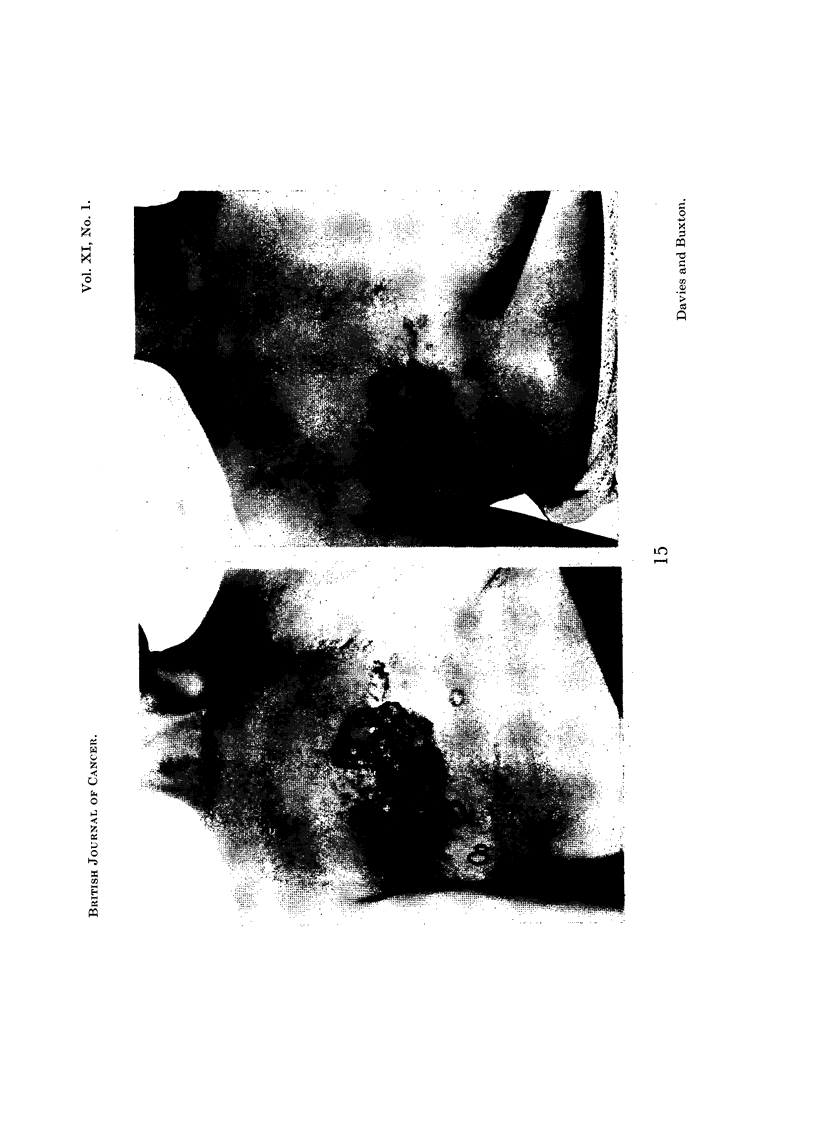

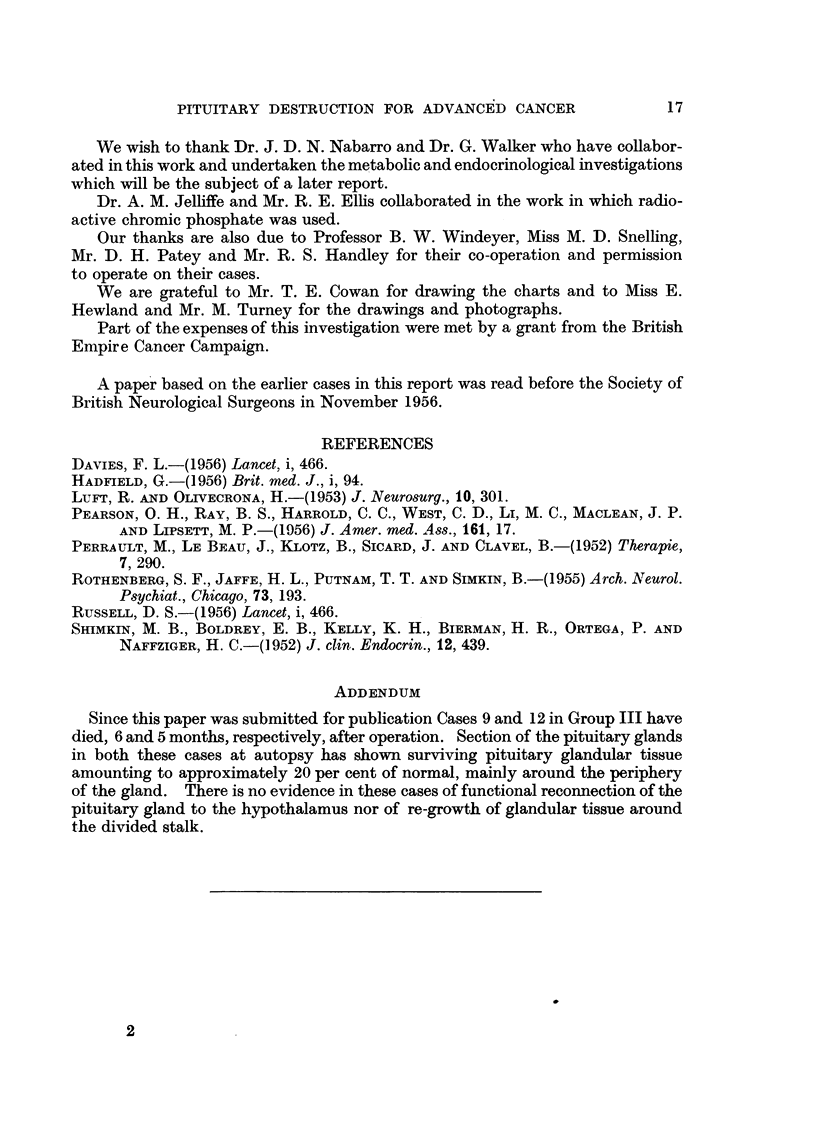

